# Pharmacokinetic and Permeation Studies in Rat Brain of Natural Compounds Led to Investigate Eugenol as Direct Activator of Dopamine Release in PC12 Cells

**DOI:** 10.3390/ijms24021800

**Published:** 2023-01-16

**Authors:** Barbara Pavan, Anna Bianchi, Giada Botti, Luca Ferraro, Maria Chiara Valerii, Enzo Spisni, Alessandro Dalpiaz

**Affiliations:** 1 Department of Neuroscience and Rehabilitation—Section of Physiology, University of Ferrara, via L. Borsari 46, I-44121 Ferrara, Italy; 2 Center for Translational Neurophysiology of Speech and Communication (CTNSC), Italian Institute of Technology (IIT), via Fossato di Mortara 19, I-44121 Ferrara, Italy; 3 Department of Chemical, Pharmaceutical and Agricultural Sciences, University of Ferrara, via Fossato di Mortara 19, I-44121 Ferrara, Italy; 4 Department of Life Sciences and Biotechnology, University of Ferrara and LTTA Center, via Fossato di Mortara 19, I-44121 Ferrara, Italy; 5 Targeting Gut Disease Srl, Viale Fanin 48, I-40136 Bologna, Italy; 6 Department of Biological, Geological and Environmental Sciences, Alma Mater Studiorum-University of Bologna, Via Selmi 3, I-40126 Bologna, Italy

**Keywords:** essential oils, eugenol, D-limonene, cinnamaldehyde, HPLC-UV, pharmacokinetic studies, central nervous systems, dopamine release, cell viability, PC12 cells

## Abstract

Eugenol, cinnamaldehyde and D-limonene, the main components of natural essential oils, are endowed with antioxidant and anti-inflammatory properties which allow them to induce beneficial effects on intestinal, cardiac and neuronal levels. In order to characterize their pharmacokinetic profiles and aptitude to permeate in the central nervous system after intravenous and oral administration to rats, new analytical procedures, easily achievable with HPLC-UV techniques, were developed. The terminal half-lives of these compounds range from 12.4 ± 0.9 (D-limonene) and 23.1 ± 1.6 min (cinnamaldehyde); their oral bioavailability appears relatively poor, ranging from 4.25 ± 0.11% (eugenol) to 7.33 ± 0.37% (cinnamaldehyde). Eugenol evidences a marked aptitude to permeate in the cerebrospinal fluid (CSF) of rats following both intravenous and oral administrations, whereas cinnamaldehyde appears able to reach the CSF only after intravenous administration; limonene is totally unable to permeate in the CSF. Eugenol was therefore recruited for in vitro studies of viability and time-/dose-dependent dopamine release in neuronal differentiated PC12 cells (a recognized cellular model mimicking dopaminergic neurons), evidencing its ability to increase cell viability and to induce dopamine release according to a U-shaped time-course curve. Moreover, concentration-response data suggest that eugenol may induce beneficial effects against Parkinson’s disease after oral administration.

## 1. Introduction

Essential oils (EOs) are a complex mixture of volatile compounds produced as secondary metabolites by aromatic plants in order to obtain their defense from external agents, such as bacteria, fungi and viruses [1]. Some of the major components of EOs have been recently identified as attractive molecules for the development of new drugs with action against microbial pathogens or to counter the oxidative and inflammatory processes involved in noncommunicable diseases [2,3]. This study is focused on eugenol, cinnamaldehyde and D-limonene, that constitute the main components of *Syzygium aromaticum* (clove), *Cinnamomum* spp. and *Citrus sinensis* EOs, respectively [1]. The antioxidant and anti-inflammatory properties of these compounds have been previously evidenced by their ability to modulate signaling pathways such as those involving nitric oxide (NO) synthesis or nuclear factor kappa-light-chain-enhancer of activated B cells (NF-*κ*B), with related downregulation of proinflammatory cytokines [3,4,5]. The antioxidant and anti-inflammatory properties of these compounds can contribute to their beneficial effects against intestinal diseases at the level of mucosal barrier or microbiota ecology [6,7,8], but also against ischemic phenomena of the heart [9,10,11], or against neurodegeneration [4,12,13]. Taking into account the significant therapeutic opportunities that these compounds can offer in the human body, first of all, we investigated their concentration profiles during their time in the bloodstream and in the central nervous system (CNS) of rats, after intravenous and oral administration. For this purpose, we developed analytical procedures easily achievable with HPLC-UV techniques after purification of blood samples; moreover, the investigation on CNS concentrations of the compounds was performed by withdrawal of cerebrospinal fluid (CSF) samples at programmed time points from the same rats used for blood sampling, allowing to couple blood and CSF concentrations of the compounds.

In addition, it is well known the existence of bidirectional communication pathways where the gut and the brain influence each other along the so-called “gut–brain axis”. This communication route involves the gut microbiota, which plays a pivotal role in maintaining local, systemic and gut–brain homeostasis [14]. EOs may act as modulators of the intestinal microbiota [1] and their main components are considered agents able to alleviate the effects related to microbiota dysbiosis [6,7,15]. This ability, via the gut–brain axis, may be reflected by the appropriate homeostasis of the brain. Unfortunately, many components of EOs can be toxic, even when their doses are slightly larger than those at which they may exert their therapeutic effects [1].

Thus, in order to prevent potential systemic toxicity, it should be important to reduce the intestinal absorption of these compounds, to propose their daily administration as a strategy to preserve a balanced gut microbiota and to maintain a healthy status. The analytical procedures proposed in this paper were then applied to oral bioavailability studies which were also extended to formulations designed to reduce the intestinal absorption of eugenol, cinnamaldehyde and D-limonene after their oral administration to rats.

To summarize, the goals of this study were to define (*i*) the pharmacokinetic profile of eugenol, cinnamaldehyde and D-limonene following their intravenous administration to rats; (*ii*) the bioavailability of these compounds after their oral administration (as they are or formulated); (*iii*) their aptitude to permeate in CSF from the bloodstream, by evaluating the ratio (R) of their concentration between CSF and the blood of rats at same times after administration; finally, (*iv*) we tried to analyze the possible effects of the EO components that easily permeate into the brain by studying in vitro their potential direct effect on cell viability and dopamine release. It is indeed known that EO components can exert neuroprotective effects on rat models of Parkinson’s disease (PD) [16]. Therefore, we used PC12 cell line, a cellular model recognized as suitable to mimic dopaminergic neurons [17], to evaluate the ability of EO components to modulate dopamine release.

## 2. Results and Discussion

### 2.1. Formulations and Doses for the Administration of Eugenol, Cinnmaldehyde and D-Limonene

In a first set of experiments, we performed a comparison among eugenol, cinnamaldehyde and D-limonene by evaluating their (*i*) kinetic elimination from the bloodstream, (*ii*) oral bioavailability and (*iii*) potential ability to permeate into the CNS after intravenous and oral administrations to rats. The administered doses of compounds and the related formulations were chosen in order to both avoid toxicity to rats and to obtain quantifiable amounts of the three compounds, via HPLC-UV, in the bloodstream and CSF samples. Literature data report that intravenous administrations of eugenol doses up to 60 mg/kg induce anesthetic effects in rats, without relevant side effects [18]. A dose of 20 mg/kg of eugenol was therefore chosen for the intravenous administration to rats, also taking into account that, in this study, the rats were previously anesthetized before its administration. Eugenol was formulated as a stable dispersion of fine droplets in a saline solution. The stability of the emulsion was obtained in the presence of cremophor (density = 0.961 g/mL), whose volumetric ratio was of 3:1 with respect to eugenol. Under these conditions the rats received a cremophor dose of 54.4 mg/kg, i.e., one order of magnitude lower than the maximum dosages without toxicity [19]. A eugenol dose of 500 mg/kg was chosen for the oral administration to rats, as its oral LD_50_ value is about 2 g/kg [20]. Eugenol (free compound) was formulated as a solution in corn oil, as previously described by other authors for the oral administration of main components of EOs [21,22]. Moreover, eugenol was included in oral formulations designed to promote its retention in the intestinal environments, by absorbing this chemical compound on vegetal fibers (EU-GN formulation; 20% *w*/*w* of eugenol), or by complexing with β-cyclodextrins (EU-CD formulation; 30 mg/mL eugenol), or by microencapsulation into soy lecithin micelles (EU-SL formulation; 390 mg/mL eugenol).

Concerning cinnamaldehyde, a 25 mg/kg dose intravenously administered to rats is known to induce toxicity, whereas 30 mg/kg appears lethal [22]. A dose of 20 mg/kg of cinnamaldehyde was therefore chosen for the intravenous administration to rats. Cinnamaldehyde was formulated as a stable dispersion of fine droplets in a saline solution. The stability of the emulsion was obtained in the presence of both cremophor (density = 0.961 g/mL), whose volumetric ratio was of 1:1 with respect to cinnamaldehyde, and ethanol (10% with respect to the total volume of the formulation). Under these conditions the rats received a cremophor dose of 18.3 mg/kg, widely below the maximum dosages without toxicity [19]. A cinnamaldehyde dose of 400 mg/kg was chosen for the oral administration to rats, as its oral LD_50_ value is about 3 g/kg [22]. Cinnamaldehyde (free compound) was formulated as a solution in corn oil, as previously described by other authors for the oral administration of main components of EOs [21,22]. Moreover, cinnamaldehyde was included in an oral formulation designed to promote the retention of this compound in the intestinal environments, by absorbing this chemical compound on vegetal fibers (AC-GN formulation; 14% *w*/*w* of cinnamaldehyde).

Concerning D-limonene, the dose chosen for intravenous administration to rats was 100 mg/kg, below its LD_50_ value [23]. In this case, the very high lipophilicity of this compound did not allow to obtain stable emulsions in saline solution, despite the presence of cremophor and ethanol. D-Limonene was therefore intravenously administered as pure compound, as previously reported [24]. A dose of 200 mg/kg was chosen for the oral administration of D-limonene to rats, taking into account that its LD_50_ value is about 4 g/kg [25]. D-Limonene (free compound) was formulated as a solution in corn oil, as previously described by other authors for the oral administration of the main components of EOs [21,22]. Moreover, D-limonene was included in an oral formulation designed to promote the retention of this compound in the intestinal environments, by absorbing this chemical compound on vegetal fibers (LM-CA formulation; 17% *w*/*w* D-limonene) [8].

### 2.2. Intravenous Administration of Eugenol, Cinnamaldehyde and D-Limonene

The doses and modalities chosen for the intravenous administration of eugenol, cinnamaldehyde and D-limonene allowed to obtain, at the end of their infusion, concentrations (C_0_) in the bloodstream of rats of around 20 μg/mL for the three compounds. The C_0_ values detected for these compounds were about 200–300-fold higher than their lower limit of calibration in the whole blood of rats. The extraction procedures and the HPLC-UV methods can be therefore proposed as novel and simple analytical tools for pharmacokinetic studies of eugenol, cinnamaldehyde and D-limonene. Acetonitrile was chosen as a protein denaturing agent when blood samples were prepared for analysis of eugenol and D-limonene, whereas the protein denaturation was performed in acidic environments in the case of cinnamaldehyde extraction (see below).

**Figure 1:**
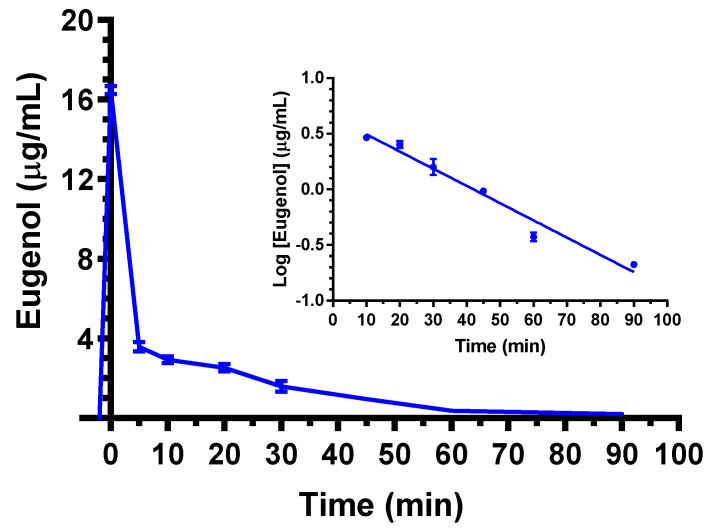
Elimination profile of eugenol after 5 mg intravenous infusion to rats (20 mg/kg). Data are expressed as the mean ± SE of four independent experiments. The elimination showed a relatively high rate within 5 min from the end of infusion; then, from 10 min after the end of infusion, it followed an apparent first order kinetics confirmed by the semilogarithmic plot reported in the inset (*n* = 6, *r* = 0.982, *p* < 0.001). The terminal half-life of eugenol was calculated to be 19.4 ± 2.1 min.

As evidenced in Figure 1, the eugenol concentration in the rat bloodstream was 16.5 ± 0.2 μg/mL at the end of 5 mg (20 mg/kg) dose intravenous infusion. This value rapidly decreased to 3.6 ± 0.2 μg/mL and 2.9 ± 0.1 μg/mL after 5 and 10 min, respectively; then, the eugenol concentration declined over time with an apparent first order kinetic confirmed by the linearity of the semilogarithmic plot reported in the inset of Figure 1 (*n* = 6, *r* = 0.982, *p* < 0.001), showing a terminal half-life (t_1/2_) of 19.4 ± 2.1 min, that was obtained by the kinetic elimination constant (k_el_) value of 0.036 ± 0.002 min^−1^. The area under the time/concentration curve (AUC) value in the bloodstream obtained by eugenol infusion from its starting time to infinity was 174.8 ± 3.1 μg·mL^−1^·min; the clearance (Cl, as a mean of the two kinetics of elimination) was calculated as 114 ± 2 mL·min^−1^·kg^−1^ and the mean distribution volume (V_d_) as 3212 ± 247 mL/Kg (Table 1).

**Figure 2:**
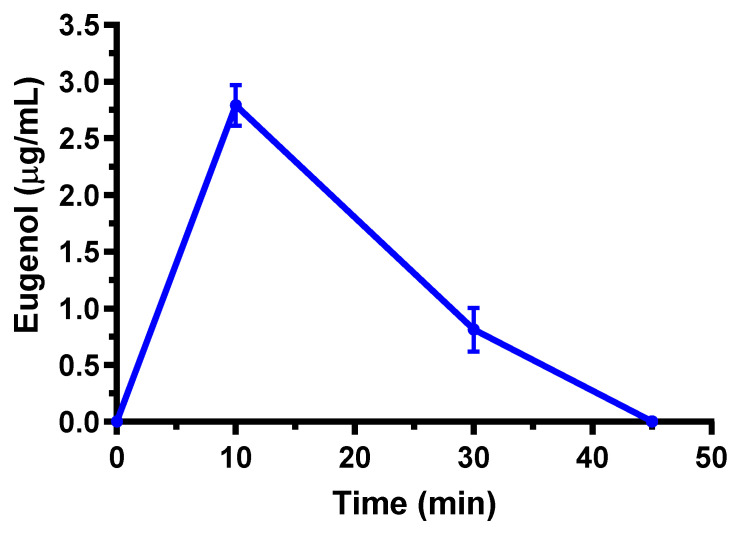
Eugenol concentrations (μg/mL) detected in the CSF of rats after intravenous administration of a 5 mg (20 mg/kg) dose. Data are expressed as the mean ± SE of four independent experiments.

The pharmacokinetic behavior of eugenol is in line with that detected in previous studies [18]. In particular, it is confirmed the relatively fast reduction of eugenol amounts in the bloodstream within a few minutes from the administration, followed by a second phase characterized with a t_1/2_ value around 13–20 min. Moreover, the Cl and V_d_ values appear markedly higher than those compatible with renal or biliary elimination of eugenol and the actual volume of rats, respectively. On the basis of these data, other authors previously hypothesized a rapid distribution of eugenol to peripheral tissues [18]. According to this hypothesis, a marked aptitude of eugenol to permeate in the CNS from the bloodstream, following its intravenous administration, was observed. Indeed, as reported in Figure 2, eugenol was detected in the rat CSF showing a maximum concentration (C_max_) of 2.79 ± 0.18 μg/mL at 10 min (T_max_) after the end of infusion; then its concentrations decreased to zero within 45 min. The AUC value in the CSF obtained by eugenol infusion from the end of its infusion to 45 min was 56.1 ± 4.2 μg·mL^−1^·min. The ratio of the eugenol concentration between CSF and the bloodstream (R) at 10 min from the end of infusion (T_max_ of CSF) was calculated as 0.96 ± 0.08, indicating, at this time, similar concentration values of this compound in both the blood and CSF of rats.

**Figure 3:**
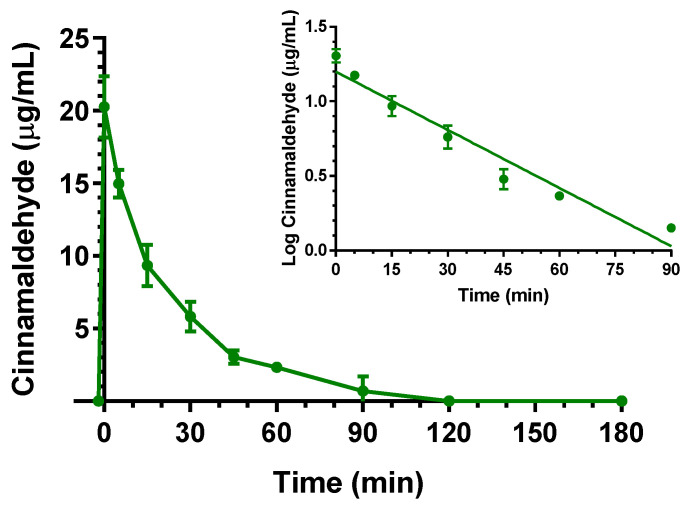
Elimination profile of cinnamaldehyde after 5 mg (20 mg/kg) intravenous infusion to rats. Data are expressed as the mean ± SE of four independent experiments. The elimination followed an apparent first order kinetics, confirmed by the semilogarithmic plot reported in the inset (*n* = 7, *r* = 0.976, *p* < 0.001). The half-life of cinnamaldehyde was calculated to be 23.1 ± 1.6 min.

The intravenous administration to rats of 5 mg (20 mg/kg) of cinnamaldehyde allowed to obtain the profile reported in Figure 3. In particular, at the end of infusion, the cinnamaldehyde concentration in the rat bloodstream was 20.3 ± 1.5 mg/mL. Other authors have previously reported C_0_ values of about 0.5 μg/mL, following the intravenous administration of the same dose (20 mg/kg) of cinnamaldehyde to rats [26,27]. This strong discrepancy may be due to differences in extraction procedures between this study and the previously published ones. In particular, it is known that, as a typical reactive aldehyde, cinnamaldehyde can conjugate with various amino acids or proteins through amino or thiol groups to form Schiff’s bases and thiol conjugates [22,28]. Acetonitrile can be considered a strong denaturing agent of proteins where they are practically insoluble [29], so the resulting Schiff base products appear unextractable with acetonitrile. This solvent was previously chosen for sample preparation [26], even if the free amounts of cinnamaldehyde detectable in the blood were very poor, requiring gas chromatography–mass spectrometry techniques for their quantification [26]. On the other hand, it is known that Schiff’s bases can be hydrolyzed to release aldehydes under acid conditions [22]. For this reason, we chose to perform the denaturation of rat blood proteins in acidic environments (instead of acetonitrile), then to extract the released cinnamaldehyde with ethyl acetate. This strategy allowed us to obtain recovery cinnamaldehyde percentages of about 45% and to quantify the compound blood concentrations lower than 0.07 μg/mL by HPLC-UV techniques. The use of formaldehyde as a competitive suppressor of Schiff base formation was proposed as an alternative method to the acidic denaturation of blood proteins. This strategy allowed us to quantify by HPLC-UV cinnamaldehyde C_0_ values of about 20 mg/mL following the administration of a 25 mg/kg dose [22].

As reported in Figure 3, the cinnamaldehyde C_0_ value decreased over time with an apparent first order kinetic, confirmed by the linearity of the semilogarithmic plot reported in the inset of Figure 3 (*n* = 7, *r* = 0.976, *p* < 0.001) and showing a t_1/2_ of 23.1 ± 1.6 min, that was obtained by the k_el_ value of 0.0299 ± 0.0013 min^−1^. The cinnamaldehyde in vivo profile can be attributed to both its oxidation in the blood, leading cinnamic acid, and to conjugation with proteins of the body, then followed by its release [22]. The AUC value in the bloodstream obtained by cinnamaldehyde infusion from its starting time to infinity was 506.0 ± 22.3 μg·mL^−1^·min; the Cl was calculated as 39.5 ± 1.7 mL·min^−1^ kg^−1^ and the V_d_ as 1320 ± 190 mL/kg (Table 1).

**Figure 4:**
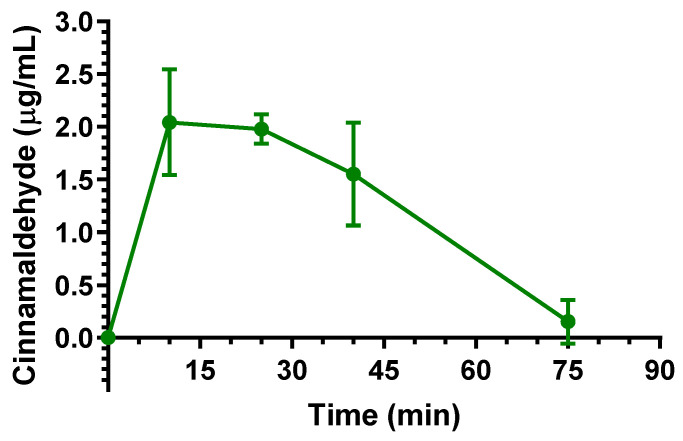
Cinnamaldehyde concentrations (μg/mL) detected in the CSF of rats after intravenous administration of a 5 mg (20 mg/kg) dose. Data are expressed as the mean ± SE of four independent experiments.

In comparison with eugenol, the Cl and V_d_ values of cinnamaldehyde suggest a lower aptitude of this compound for peripheral distribution. Nevertheless, following the intravenous administration, cinnamaldehyde evidenced the ability to permeate in the CNS from the bloodstream. Indeed, as reported in Figure 4, cinnamaldehyde was detected in the CSF of rats showing C_max_ of 2.04 ± 0.36 μg/mL at 10 min (T_max_) after the end of infusion, then its concentrations decreased to values near to zero within 75 min. The AUC value in the CSF obtained by cinnamaldehyde from the end of its infusion to 75 min was 96.67 ± 10.96 μg·mL^−1^·min. The ratio of the cinnamaldehyde concentration between CSF and the bloodstream (R) at 10 min from the end of infusion (T_max_ of CSF) was estimated, based on the pattern reported in Figure 3, as 0.156 ± 0.038. This value, strongly lower than that found for eugenol at similar conditions (R = 0.96), seems in accordance with the lower aptitude of cinnamaldehyde to distribute itself in the body out of the bloodstream; however, a comparison of the CSF profiles and AUC values (Table 2) indicates a prolonged permanence of cinnamaldehyde in central environments in comparison to eugenol.

**Figure 5:**
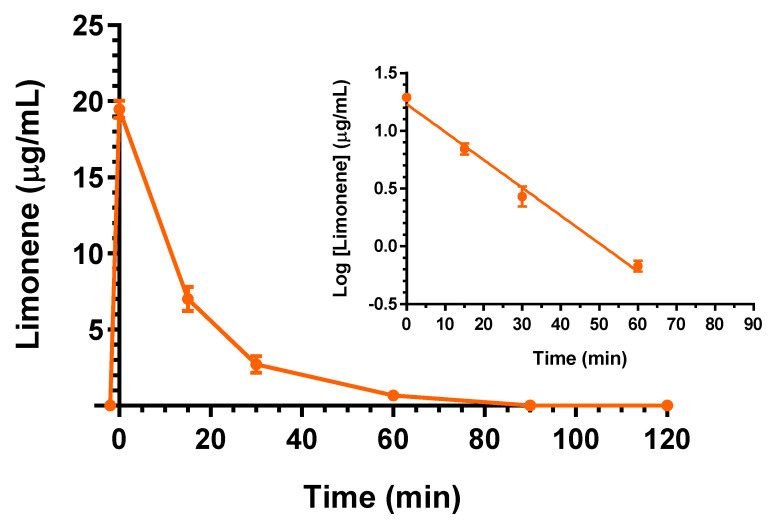
Elimination profile of D-limonene after 25 mg (100 mg/kg) intravenous infusion to rats. Data are expressed as the mean ± SE of four independent experiments. The elimination followed an apparent first order kinetics, confirmed by the semilogarithmic plot reported in the inset (*n* = 4, *r* = 0.995, *p* < 0.01). The half-life of D-limonene was calculated to be 12.4 ± 0.9 min.

The intravenous administration to rats of 25 mg (100 mg/kg) of D-limonene allowed us to obtain the profile reported in Figure 5. In particular, at the end of infusion the D-limonene concentration in the rat bloodstream was 19.5 ± 0.4 mg/mL. This value decreased over time with an apparent first order kinetic confirmed by the linearity of the semilogarithmic plot reported in the inset of Figure 5 (*n* = 4, *r* = 0.995, *p* < 0.01), showing a t_1/2_ of 12.4 ± 0.9 min, that was obtained by the k_el_ value of 0.0555 ± 0.0040 min^−1^. The t_1/2_ value appears in good agreement with that previously detected, following 200 mg/kg D-limonene intravenous administration [24]. This dosage was higher than D-limonene LD_50_ for the rats (120 mg/kg [23]), and under those conditions, the C_0_ was reported higher than 80 μg/mL, and the D-limonene profile was characterized by a terminal t_1/2_ of 280 min [24]. According to the profile reported in Figure 5, the AUC value in the bloodstream obtained by D-limonene infusion from its starting time to infinity was 352.2 ± 13.1 μg·mL^−1^·min; the Cl was calculated as 283.9 ± 10.4 mL·min^−1^·kg^−1^ and the V_d_ as 5516 ± 558 mL/kg (Table 1). These last values indicate the strong aptitude of D-limonene to permeate in extravascular compartments of the body, as previously reported by other authors [24,30]. On the other hand, following the intravenous administration, D-limonene did not evidence the ability to permeate in the CNS from the bloodstream. Indeed, this compound was not detected in the CSF of rats within 90 min after the end of infusion.

Table 1 summarizes the pharmacokinetics values referred to the bloodstream obtained by the intravenous administration to rats of eugenol, cinnamaldehyde and D-limonene, whereas Table 2 summarize the pharmacokinetic values referred to CSF.

### 2.3. Oral Administration of Eugenol, Cinnamaldehyde and D-Limonene

**Figure 6:**
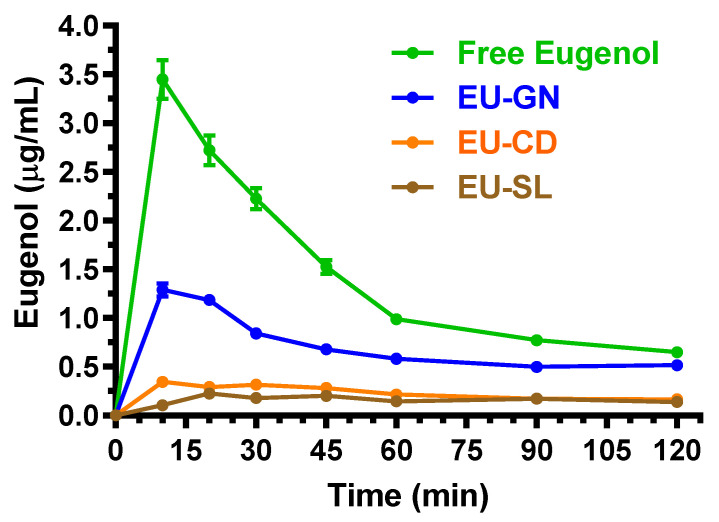
Blood eugenol concentrations (μg/mL) within 120 min after oral administration of 500 mg/kg doses to rats. Data are expressed as the mean ± SE of four independent experiments. The oral formulations consisted of dissolved eugenol in corn oil (free eugenol), or eugenol adsorbed on vegetal fibers (EU-GN) or complexed with cyclodextrins (EU-CD) or included with soy lecithin (EU-SL).

The doses chosen for the oral administration of eugenol, cinnamaldehyde and D-limonene in their free forms allowed us to obtain C_max_ values in the bloodstream of rats ranging between about 2 and 8 μg/mL. The inclusion of the compounds in the oral formulations based on vegetal fibers, cyclodextrins or lipids induced a significant decrease of their absorption in the bloodstream. In particular, Figure 6 reports the concentration profiles of eugenol in the bloodstream of rats after the oral administration of 500 mg/kg in the free form (solution in corn oil) or included in the EU-GN (adsorbed on vegetal fibers), EU-CD (cyclodextrin complex) and EU-SL (soy lecithin inclusion) formulations.

After the oral administration of eugenol in the free form (corn oil solution), the higher concentration in the rat bloodstream was obtained at 10 min (T_max_) with a value of 3.4 ± 0.2 μg/mL (C_max_). These data are in line with those obtained by previous pharmacokinetic studies following the oral administration of eugenol to rats, where an oral dose of 40 mg/kg allowed us to obtain a C_max_ value of about 0.25 μg/mL at 15 min [31]. The AUC value of the eugenol profile, here calculated from the time 0 to infinity, was 185.7 ± 3.1 μg·mL^−1^·min, which allowed us to obtain the absolute bioavailability (F) value of 4.25 ± 0.11%. This result indicates a relatively poor aptitude of eugenol to be absorbed in the bloodstream of rats after its oral administration in the free form.

**Figure 7:**
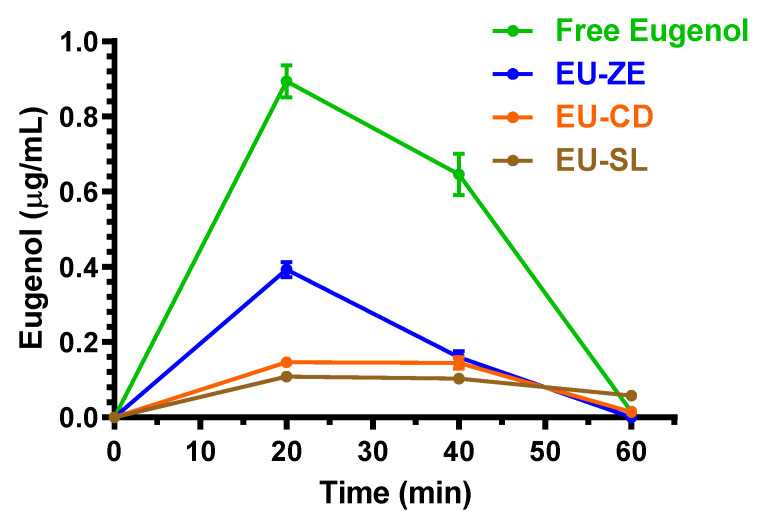
CSF eugenol concentrations (μg/mL) within 60 min after oral administration of 500 mg/kg doses to rats. Data are expressed as the mean ± SE of four independent experiments. The oral formulations consisted of dissolved eugenol in corn oil (free eugenol), or eugenol adsorbed in vegetal fibers (EU-GN) or complexed with cyclodextrins (EU-CD) or included with soy lecithin (EU-SL).

Despite the relatively poor oral F value of eugenol, its presence was however quantifiable in the CSF of rats after the oral administration of 500 mg/kg in the free form. Indeed, as reported in Figure 7, the C_max_ value of eugenol in CSF was 0.89 ± 0.06 μg/mL at 20 min (T_max_); the CSF concentration profile allowed us to obtain an AUC value of 30.97 ± 2.18 μg·mL^−1^·min. These results confirm the high aptitude of eugenol to permeate in the CNS from the bloodstream, as evidenced by the R value (Table 2) of about 1 as referred to the intravenous administration, indicating a CSF eugenol concentration at T_max_ of its profile (Figure 2), similar to that detected in the bloodstream.

The inclusion of eugenol in the oral formulations EU-GN, EU-CD and EU-SL induced a further decrease of its oral bioavailability (Figure 6); however, the oral administration to rats of these formulations (eugenol dose = 500 mg/kg) allowed us to quantify this compound in the CSF, as reported in Figure 7. In particular, following the oral administration of eugenol as EU-GN and EU-CD formulations, the higher concentrations in the rat bloodstream were obtained at 10 min (T_max_) with C_max_ values of 1.29 ± 0.10 μg/mL and 0.35 ± 0.05 μg/mL, respectively (Figure 6). The AUC values of the eugenol profiles, calculated from the time 0 to infinity, were 95.6 ± 1.5 μg·mL^−1^·min and 31.6 ± 1.7 μg·mL^−1^·min, respectively, which allowed us to obtain the F values of 2.19 ± 0.05% and 0.72 ± 0.04% for EU-GN and EU-CD oral formulations, respectively. These values were significantly lower (*p* < 0.001) than the F value obtained by oral administration of eugenol in the free form. Following the oral administration of eugenol as EU-SL formulations, the higher concentration in the rat bloodstream was obtained at 20 min (T_max_) with a C_max_ value of 0.23 ± 0.01 μg/mL. The AUC value of the eugenol profile, calculated from the time 0 to infinity, was 29.2 ± 0.7 μg·mL^−1^·min, which allowed us to obtain the F value of 0.67 ± 0.02%, significantly lower (*p* < 0.001) in comparison with the F value obtained by the oral administration of eugenol in the free form. According to these results, the relative bioavailability (RB) values obtained by oral administration of EU-GN, EU-CD and EU-SL were 51.5 ± 1.1, 17.0 ± 1.0 and 15.7 ± 0.5 in comparison with the oral administration of eugenol in the free form.

Despite the significant decrease of oral bioavailability induced by the EU-GN, EU-CD and EU-SL, it was anyway possible to quantify eugenol in the CSF of rats, as reported in Figure 7. In particular, the C_max_ values of eugenol in CSF were obtained at 20 min (T_max_), being 0.39 ± 0.03 μg/mL, 0.146 ± 0.002 μg/mL and 0.110 ± 0.006 μg/mL for EU-GN, EU-CD and EU-SL formulations, respectively; the CSF concentration profiles allowed us to obtain AUC values of 11.05 ± 0.69 μg·mL^−1^·min, 5.96 ± 0.31 μg·mL^−1^·min and 5.69 ± 0.09 μg·mL^−1^·min, respectively. All the values were significantly lower (*p* < 0.001) than those obtained by the oral administration of eugenol in the free form.

**Figure 8:**
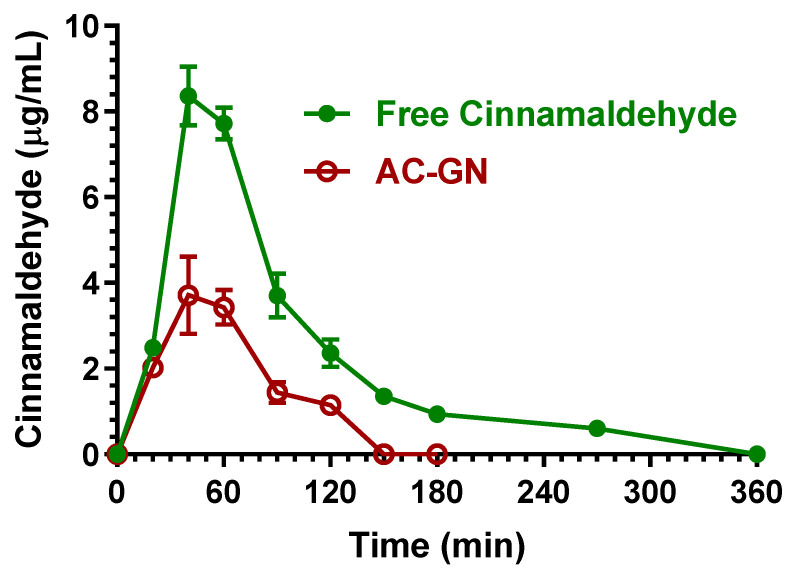
Blood cinnamaldehyde concentrations (μg/mL) within 360 min after oral administration of 400 mg/kg doses to rats. Data are expressed as the mean ± SE of four independent experiments. The oral formulations consisted of dissolved cinnamaldehyde in corn oil (free Cinnamaldehyde) or cinnamaldehyde adsorbed in vegetal fibers (AC-GN).

Figure 8 reports the concentration profiles of cinnamaldehyde in the bloodstream of rats after the oral administration of 400 mg/kg in the free form (solution in corn oil) or included in the AC-GN (adsorbed on vegetal fibers) formulation.

About the oral administration of cinnamaldehyde in the free form (corn oil solution), the higher concentration in the rat bloodstream was obtained at 40 min (T_max_) with a C_max_ value of 8.36 ± 0.49 μg/mL. The AUC value of the cinnamaldehyde profile, calculated from the time 0 to infinity, was 742.7 ± 18.4 μg·mL^−1^·min, which allowed us to obtain the F value of 7.33 ± 0.37%, a value in agreement with those previously indicated by other authors (less than 20%) [22,26]. This result indicates a relatively poor aptitude of cinnamaldehyde to be absorbed in the bloodstream of rats after its oral administration in the free form, even if the F value appears higher with respect to eugenol (F = 4.25 ± 0.11%). Following oral administration, cinnamaldehyde was not detectable in the CSF of rats within 90 min, despite the F value of this compound being higher than that of eugenol. This behavior appears consistent with the lower aptitude of cinnamaldehyde to permeate in the CNS in comparison with eugenol, as indicted by the R values (0.16 ± 0.04 for cinnamaldehyde, 0.96 ± 0.08 for eugenol, Table 2). Moreover, it should be considered that oral administration allowed us to obtain cinnamaldehyde concentrations in the bloodstream that were sensibly lower than the higher values obtained after cinnamaldehyde intravenous administration, with consequent possible increase of the percentages of compound involved in the formation of Schiff’s bases and thiol conjugates [22,28] unavailable to permeate in the CSF.

The absorption of cinnamaldehyde into vegetal fibers (AC-GN) induced a further decrease of its oral bioavailability (Figure 8). In particular, the higher concentration in the rat bloodstream was obtained at 40 min (T_max_) with a C_max_ of 3.71 ± 0.64 μg/mL. The AUC value of the cinnamaldehyde profile, calculated from the time 0 to infinity, was 278.3 ± 15.9 μg·mL^−1^·min, indicating an F value of 2.75 ± 0.19%, significantly lower (*p* < 0.001) than the F value obtained by oral administration of cinnamaldehyde in the free form. According to these results, the RB values obtained by oral administration of AC-GN was 37.5 ± 3.2%. Also following the oral administration of AC-GN formulation, cinnamaldehyde was not detectable in the CSF of rats.

**Figure 9:**
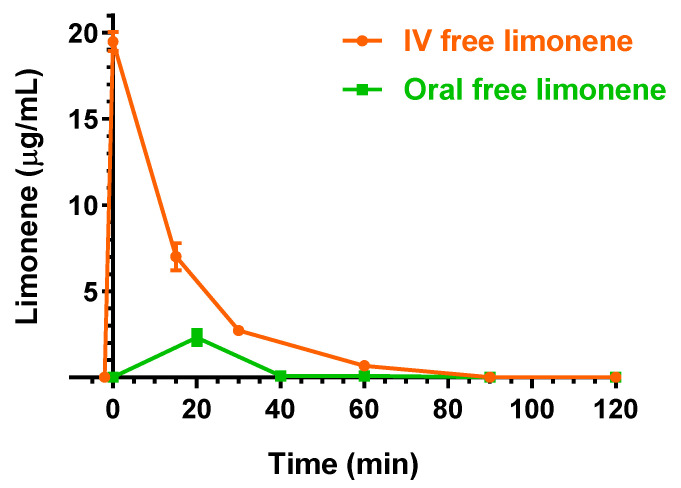
Blood D-limonene concentrations (μg/mL) within 120 min after intravenous (IV) and oral administration in the free form of 100 mg/kg and 200 mg/kg doses, respectively, to rats. Data are expressed as the mean ± SE of four independent experiments.

Figure 9 reports a comparison of the D-limonene profiles obtained by the intravenous (IV) and oral administration of 100 mg/kg and 200 mg/kg doses, respectively, of the drug in free form.

Following the oral administration, the higher D-limonene concentration was obtained at 30 min (T_max_) with a C_max_ value of 2.31 ± 0.44 μg/mL. Other authors reported C_max_ values of about 11 μg/mL or 0.3 μg/mL following the oral administration of D-limonene at 200 mg/kg [24] or 75 mg/kg doses [32], respectively. The AUC value of the D-limonene profile, calculated from the time 0 to infinity, was 49.6 ± 6.7 μg·mL^−1^·min, indicating an F value of 7.04 ± 0.96%, lower than that reported by a previous study [24]. Recently, an improvement of oral bioavailability of D-limonene was proposed by formulating a self-micro-emulsifying drug delivery system [32]. According to our results, the oral absolute bioavailability of cinnamaldehyde and D-limonene appear similar to each other. As evidenced for the intravenous administration, the oral administration of free D-limonene did not allow its detection in the CSF of rats. Finally, the oral administration of the same dose of D-limonene (200 mg/kg) included in LM-CA formulation did not allow us to detect any amounts of this compound neither in the bloodstream [8] nor in the CSF of rats.

The pharmacokinetic values referred to the bloodstream and CSF, obtained by the oral administration to rats of eugenol, cinnamaldehyde and D-limonene are summarized in Table 3 and Table 4, respectively.

The results reported in Table 4 confirm the extraordinarily high aptitude of eugenol to permeate in the CSF of rats from the bloodstream. Among the compounds analyzed, only eugenol was indeed able to reach the CSF following any type of administration, whereas cinnamaldehyde showed this ability only after intravenous administration (with an R value sensibly lower than that of eugenol, Table 2), and D-limonene was never detected in the CSF of rats.

About the oral availability, all compounds evidenced relatively poor values (Table 3), ranging from about 4% to 7%, differently from geraniol (one of the major components of EO obtained by plants of the genus *Cymbopogon* or *Pelargonium* [1]), whose oral bioavailability appeared about 90% [33]. For this compound an influx active system was evidenced by in vitro studies performed on a model of the human intestinal wall [33]. Oral formulations, obtained by geraniol absorption on ginger fibers, induced a drastic reduction of its bioavailability to about 16% [33].

The formulations based on vegetal fibers allowed us to reduce the oral F values of eugenol, cinnamaldehyde and D-limonene to about 2%, 3% and 0, respectively; eugenol further decreased its F values to about 0.5% when included on formulations based on cyclodextrins or solid lipids. These extremely low F values suggest a potential use of these formulations for chronic daily oral administrations deputed to the microbiota maintenance, without producing a significant impact to the other compartments of the body. The components of EOs are indeed known as potent modulators of gut inflammation and microbiota ecology [34]. It is important to consider that brain neurochemistry is affected by gut microbiota according to the so called “gut–brain axis”; in particular, the two systems cooperate in a bidirectional way, both to regulate the absorption of nutrients in the intestine by CNS and to maintain the homeostasis of the CNS by regulating the permeability of the intestinal barrier [14]. As a result, gut microbiota is critically important for the appropriate maintenance of brain function. In view of the promising pharmacokinetic properties, it will be interesting to evaluate, in further studies, the effects of chronic daily oral administrations of the tested formulations of EO components on gastrointestinal microbiota composition.

Based on the pharmacokinetic results, eugenol showed the best ability to permeate into the CNS from the bloodstream, compared to the other compounds, after both intravenous and oral administration in rats. Therefore, also considering the latter topics, eugenol was selected for subsequent studies carried out in the PC12 cell line, a neuronal-like cell model recognized as suitable to mimic dopaminergic neurons [17]. These experiments were aimed to predictively evaluate the potential neuroprotective effects of eugenol directly to the CNS, both in terms of their cell viability supporting effect and of dopamine release in time and concentration-dependent manner.

### 2.4. Effect of Eugenol on Cell Viability in PC12 Cells

**Figure 10:**
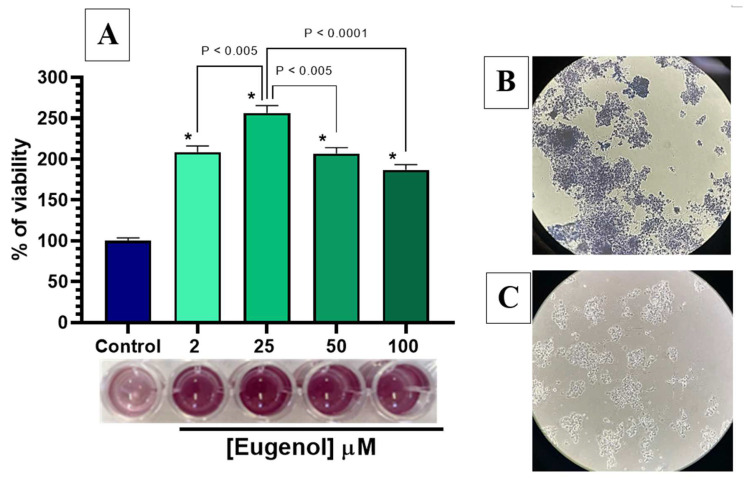
Cell viability of PC12 cells incubated with increasing concentrations of eugenol. (**A**) Data of MTT assay are presented as cell viability percentage (%) normalized to control (non-stimulated) for PC12 cells incubated with 2, 25, 50 and 100 µM eugenol. In the panel below, colorimetric results of the MTT assay are shown; (**B**) PC12 cells containing formazan crystals after incubation with 25 µM eugenol and staining with MTT; (**C**) Untreated PC12 cells differentiated to neuronal phenotype. Data are expressed as mean ± SE of three experiments run in duplicate. Data were statistically analyzed by one-way ANOVA followed by Bonferroni’s post-hoc multiple comparison test. * *p* < 0.0001 vs. control.

To investigate potential neurotoxic or neuroprotective effects of eugenol, PC12 cells differentiated to the neuronal phenotype with 100 ng/mL NGF for 14 days were exposed to increasing concentrations of eugenol (2, 25, 50 and 100 µM) for 2 h at 37 °C, followed by MTT staining for 2 h in DPBS. As reported in panel A of Figure 10, the viability assessment indicated that all tested eugenol concentrations not only had no toxic impact on the percentage of cell viability, but induced a statistically significant increase in cell viability compared to the untreated control (*p* < 0.0001), as also shown in the microwell panel of the figure. In particular, the most effective eugenol concentration was 25 µM, which increased the viability 2.5 times (*p* < 0.0001) over the untreated cell value; this concentration has also been reported as the most effective in increasing the viability of PC12 cells by resveratrol, another antioxidant natural compound [35]. Moreover, eugenol is known for its neuroprotective effects due to its anti-inflammatory and antioxidant properties, demonstrated in neuronal cell models [36]. Eugenol has further been reported to enhance the mitochondrial dehydrogenase enzymatic activity in MTT staining of cultured human periodontal ligament-derived fibroblasts, when compared with untreated cells [37]. It is worth noting that the increase of MTT signaling cannot be imputable to a higher number of cells in the different wells, since the MTT assay was performed on PC12 cells seeded at the same number for all conditions and differentiated to the proliferation-arresting neuronal phenotype after 24 h. Therefore, it remains to be investigated if the increase of mitochondrial dehydrogenase activity induced by eugenol could be due to an increased function of mitochondrial chain complexes, suggesting the enhancement of mitochondrial function as a possible mechanism underlying eugenol neuroprotective properties. As further validation of this effect, evidence of the complete membrane integrity maintained by PC12 cells after a 2-h exposure to 25 µM eugenol and stained with MTT is shown in the phase contrast microscope pictures of panel B of Figure 10, where the purple formazan crystals are fully contained within the cells, without extracellular crystals, and compared with the untreated PC12 cells of the panel C.

### 2.5. Dopamine Release evoked by Elevated Extracellular K^+^ and Eugenol in PC12 Neuronal Cell Model

**Figure 11:**
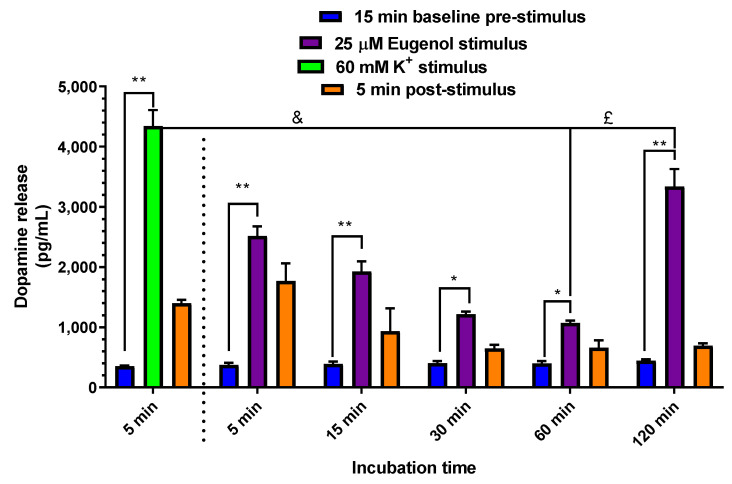
Time course of dopamine release from PC12 cells. PC12 cells were differentiated to the neuronal phenotype for 14 days, then stimulated with 60 mM K^+^ KRH at 37 °C for 5 min and with 25 µM eugenol in KRH at 37 °C for 5, 15, 30, 60 and 120 min. Each stimulation peak of release was compared to the respective pre-stimulation control or baseline release. Data are expressed as mean ± SE of three independent experiments, run in duplicate. Data were statistically analyzed by one-way ANOVA, followed by Bonferroni’s post-hoc multiple comparison test. * *p* < 0.05 stimulus vs. baseline. ** *p* < 0.001 stimulus vs. baseline. £ *p* < 0.05 60 mM K^+^ vs. eugenol 120 min. & *p* < 0.001 60 mM K^+^ vs. eugenol 60 min.

As already mentioned above, the neuronal model selected for in vitro experiments on dopamine release consisted of a PC12 cell line differentiated to a neuronal phenotype, known as suitable to synthesize, store and release dopamine [38]. This latter goal was inspired by a recent paper reporting the neuroprotective effect of eugenol exerted by its baseline antioxidant activity, combined with levodopa in a rat model of PD induced by 6-hydroxydopamine (6-OHDA) [16]. Therefore, we evaluated whether the antioxidant activity exerted by eugenol on endogenous neurotoxins in vivo could be synergistic, with a direct stimulation by eugenol on the release of dopamine. To this purpose, PC12 cells were tested for a time course of the release of dopamine evoked by 25 µM eugenol, the concentration found as the most effective in promoting cell viability in the MTT assay. As shown in Figure 11, 25 µM of eugenol induced a statistically significant increase of dopamine release, over the respective pre-treatment baseline release (393 ± 12 pg/mL) at all incubation times, with higher statistical significance at 5, 15 and 120 min (*p* < 0.001), but still significant at 30 and 60 min (*p* < 0.05). High extracellular concentrations of KCl are known to be applied in order to induce direct depolarization of the membrane potential and consequent secretion of dopamine in PC12 cells [39] and in cultures of dissociated neurons [40]. Therefore, as shown in Figure 11, a 60 mM K^+^ (5 min; i.e., a period comparable to the shortest time of eugenol stimulus) treatment was applied as a control stimulus; this stimulus enhanced the dopamine release to 4340 ± 265 pg/mL. Noteworthy, the effects of 5- to 60-min eugenol treatment were lower (*p* < 0.001) than that of 60 mM K^+^ (5 min). The effect of eugenol’s 120 min treatment was more comparable, but still statistically lower (*p* < 0.05), than that evoked by 5 min of 60 mM K^+^. Therefore, eugenol can promote a large release of dopamine after a prolonged application in cultured neurons. Actually, the time course profile of eugenol-induced dopamine release in PC12 cells showed a biphasic “U-shaped” curve, characterized by an early increase at 5 min, followed by a progressive decrease up to 60 min with the higher effect observed at 120 min. This U-shaped time-curve could indicate a hormetic or adaptive response of PC12 cells 120 min after treatment with 25 µM eugenol (Figure 11), a phenomenon where a single compound induces opposite biological responses depending on its concentration or time of exposure, as recently described by Sutou et al. [41], in in vitro cell proliferation tests. Further studies will be remarkable to investigate the cellular and molecular mechanisms underlying this U-shaped time-activity pattern of eugenol.

**Figure 12:**
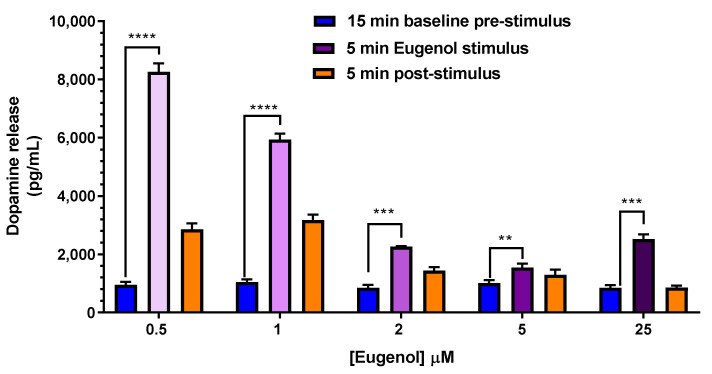
Dose-response curve of dopamine release from PC12 cells. PC12 cells were differentiated to the neuronal phenotype for 14 days, then stimulated with 0.5, 1, 2, 5 and 25 µM eugenol in KRH at 37 °C for 5 min. Each concentration was compared to the basal release of dopamine (947 ± 102 pg/mL). Data are expressed as mean ± SE of three independent experiments, run in duplicate (*n* = 6). Data were statistically analyzed by one-way ANOVA, followed by Tukey’s post-hoc multiple comparison test. **** *p* < 0.0001; *** *p* < 0.001; ** *p* < 0.01 of stimulus vs. basal pre-stimulus dopamine release.

Subsequently, a dose-response curve on dopamine release was also performed with the aim to verify if eugenol could be still efficacious at the low concentrations found in rat CSF following its oral administration, as reported above in Figure 7. Therefore, eugenol was applied at 0.5, 1, 2, 5 and 25 µM for 5 min in neuronal differentiated PC12 cells (Figure 12). Interestingly, compared to the basal release of 947 ± 102 pg/mL (*n* = 6), the highest release of dopamine was observed following the treatment with the lower concentration of eugenol (0.5 µM), corresponding to 0.08 µg/mL as found for EU-SL formulation, the lower effect still highly statistically significant vs. the basal value, was observed at 5 µM eugenol, corresponding to 0.82 µg/mL as found for free eugenol (Figure 12).

In vitro results related to the physiological effects of eugenol on cell viability, time course and dose-response relationship of dopamine release could be summarized as showing a hormetic behavior of eugenol, which could be thus speculated in protecting against neurodegenerative diseases.

## 3. Materials and Methods

### 3.1. Materials

Eugenol, cinnamaldehyde, D-limonene, carbazole, dimethyl sulfoxide (DMSO) and bovine serum albumin (BSA) were obtained from Sigma-Aldrich (Milan, Italy). Methanol, acetonitrile, ethyl acetate and water were of high-performance liquid chromatography (HPLC) grade from Carlo Erba Reagents S.A.S. (Cedex, France). Ethanol, cremophor^®^ RH 40, the nerve growth factor (NGF) and collagen IV were obtained from Merck Life Sciences Srl (Milan, Italy). RPMI-1640 (HiGlutaXL) medium, DMEM (HiGlutaXL) medium, horse serum (HS), fetal bovine serum (FBS) and cell culture vessel were furnished by Thermo-Fisher Scientific (Milan, Italy). Male Wistar rats were purchased from Charles River laboratories (Calco, Italy). Slow-release formulations consisting in (*i*) Eugenol adsorbed on vegetal fibers (EU-GN), containing 20% (*w*/*w*) of eugenol; (*ii*) Eugenol complexed with β-cyclodextrin (EU-CD), 30 mg/mL; (*iii*) Eugenol micro-encapsulated into soy lecithin micelles (EU-SL) (390 mg/mL); (*iv*) cinnamaldehyde adsorbed on vegetal fibers (AC-GN), containing 14% (*w*/*w*) of cinnamaldehyde; (*v*) D-Limonene adsorbed on vegetal fibers (LM-CA) containing 17% (*w*/*w*) of limonene were prepared and provided by Targeting Gut Disease (TGD) Srl (Bologna, Italy). Natural fibers from ginger roots (GN) were composed by 79.3 ± 6.3 (g/100 g) of insoluble part and 20.7 ± 2.4 (g/100 g) of soluble part. Soy lecithin micelles encapsulation of EO components was obtained by TGD by using a patented high efficiency procedure (patent n. WO 2011/128597, under license from Xeda International, France). 

### 3.2. In Vivo Administration of Eugenol, Cinnamaldehyde and D-Limonene

#### 3.2.1. Intravenous Administration

A saline solution (0.9% NaCl) was added to a mixture of eugenol (density = 1.06 g/mL) and Cremophor^®^ RH 40 (1:3 *v*/*v*) in order to obtain a 12.5 mg/mL eugenol emulsion. A group (*n* = 4) of male Wistar rats (200–250 g) fasted for 24 h was anesthetized during the experimental period and received 0.4 mL via a femoral intravenous infusion (rate = 0.2 mL/min; 2 min) of 12.5 mg/mL eugenol emulsion (20 mg/kg eugenol dose).

A saline solution (0.9% NaCl) was added to a mixture of cinnamaldehyde (density = 1.05 g/mL) and Cremophor^®^ RH 40 (1:1 *v*/*v*) in order to obtain a 12.5 mg/mL cinnamaldehyde emulsion in the presence of ethanol (10% of final volume). A group (*n* = 4) of male Wistar rats (200–250 g) fasted for 24 h was anesthetized during the experimental period and received 0.4 mL via a femoral intravenous infusion (rate = 0.2 mL/min; 2 min) of 12.5 mg/mL cinnamaldehyde emulsion (20 mg/kg cinnamaldehyde dose).

D-Limonene (density = 0.84 g/mL) was administered as pure compound (100 mg/kg dose). In particular, a group (*n* = 4) of male Wistar rats (200–250 g) fasted for 24 h was anesthetized during the experimental period and received a femoral intravenous infusion (rate = 15 μL/min; 2 min) of 30 μL of pure D-limonene, followed by a fast administration of saline solution (100 μL).

At the end of the infusions and at fixed time points, CSF samples (50 μL) were withdrawn and blood samples (100 μL) were collected. The CSF was withdrawn using the cisternal puncture method described by van den Berg et al. [42], which requires a single needle stick and allows the collection of serials (40–50 μL) CSF samples that are virtually blood-free [43]. A total volume of about a maximum of 150 μL of CSF/rat (i.e., three 50 μL samples/rat) was collected during the experimental session, choosing the time points (*n* = 3–5, taking into account a maximum of three collections per rat) in order to allow the restoring of the CSF physiological volume. The CSF samples (10 μL) were immediately analyzed via HPLC (see below) for the quantification of eugenol, cinnamaldehyde, or D-limonene.

Table 5 reports the modalities of the intravenous administration of the compounds to rats.

For eugenol and D-limonene analysis, the blood samples (*n* = 4) were immediately added to 200 μL of ice-cold CH_3_CN, then 100 μL of internal standard dissolved in CH_3_CN (100 μM carbazole for eugenol analysis; 100 μM GER-UDCA, obtained by the conjugation of geraniol with ursodeoxycholic acid [44], for the D-limonene analysis) were further inserted. The samples were centrifuged at 12,500× *g* for 5 min, then about 300 μL of supernatant were withdrawn and further centrifuged. Finally, 10 μL was analyzed via HPLC (see below) for eugenol or D-limonene quantification. As a control, a blood sample (100 μL) was collected by each rat before the administrations of the drugs. The control samples were immediately added to 300 μL of ice-cold CH_3_CN in the absence of internal standard, then treated as described above.

For cinnamaldehyde analysis the blood samples were hemolyzed immediately after their collection with 500 μL of ice-cold water, and then 50 μL of 10% sulfosalicylic acid and 50 μL of internal standard (100 μM carbazole dissolved in a water-methanol mixture 50:50 *v*/*v*) was added. The samples were extracted twice with 1 mL of water-saturated ethyl acetate, and, after centrifugation at 12,500× *g* for 10 min, the organic layer was reduced to dryness under a mild nitrogen stream at room temperature. Then, 200 μL of a water-acetonitrile mixture (50:50 *v*/*v*) were added, and after centrifugation, 10 μL were analyzed via HPLC (see below) for cinnamaldehyde detection. As a control, a blood sample (100 μL) was collected by each rat before the administration of the drug. The control samples were treated as described above in the absence of internal standard.

#### 3.2.2. Oral Administration

Eugenol, cinnamaldehyde and D-limonene were orally administered to rats as free drugs or included in the oral formulations EU-GN, EU-CD, EU-SL, AC-GN or LM-CA. Each type of formulation was administered to a group of four rats.

Free eugenol (125 mg) was orally administered by gavage to rats kept fasting since 24 h. In particular, 118 μL of eugenol (density = 1.06 g/mL) were dissolved in 882 μL of corn oil and administered to rats (500 mg/kg dose).

The solid EU-GN formulation (20% *w*/*w* eugenol) was mixed with palatable food in order to induce their oral intake by the rats kept fasting since 24 h. In particular, the rats assumed a dose of 125 mg of eugenol (500 mg/kg) contained in 625 mg of EU-GN.

The EU-CD formulation (30 mg/mL of eugenol) was orally administered by gavage to rats kept fasting since 24 h. In particular, 4.2 mL of EU-CD formulation were administered to rats (500 mg/kg dose).

The EU-SL formulation (390 mg/mL of eugenol) was orally administered by gavage to rats kept fasting since 24 h. In particular, 320 μL of EU-SL formulation were administered to rats (500 mg/kg dose).

Free cinnamaldehyde (100 mg) was orally administered by gavage to rats kept fasting since 24 h. In particular, 95 μL of cinnamaldehyde (density = 1.05 g/mL) were dissolved in 905 μL of corn oil and administered to rats (400 mg/kg dose).

The solid AC-GN formulation (14% *w*/*w* cinnamaldehyde) was mixed with palatable food in order to induce their oral intake by the rats kept fasting since 24 h. In particular, the rats assumed a dose of 100 mg of cinnamaldehyde (400 mg/kg) contained in 714 mg of AC-GN.

Free D-limonene (50 mg) was orally administered by gavage to rats kept fasting since 24 h. In particular, 119 μL of D-limonene (density = 0.84 g/mL) were dissolved in 881 μL of corn oil, then 500 μL of solution were administered to rats (200 mg/kg dose).

The LM-CA formulation appeared itself palatable for the rats kept fasting since 24 h. In particular, the rats assumed a dose of 50 mg of D-limonene (200 mg/kg) contained in 300 mg of LM-CA.

Table 5 summarizes the modalities of the oral administration of the compounds to rats.

After the administrations, blood (100 μL) and CSF samples (50 μL) were serially collected at fixed time points from each rat and treated as described above in order to quantify the compounds.

All in vivo experiments were performed in accordance with the European Communities Council Directive of September 2010 (2010/63/EU). Any effort has been done to reduce the number of the animals and their suffering.

#### 3.2.3. Pharmacokinetic Calculations

The in vivo half-life (t_1/2_) of eugenol, cinnamaldehyde or D-limonene, in the bloodstream of rats was calculated by nonlinear regression (exponential decay) of concentration values in appropriate time ranges after infusion and confirmed by linear regression of the log concentration values versus time (semilogarithmic plot). The area under concentration curves (AUC, μg·mL^−1^·min) related to intravenous and oral administrations of eugenol, cinnamaldehyde or D-limonene, in the bloodstream or CSF of rats were calculated by the trapezoidal method.

In particular, all the AUC values referred to the bloodstream were calculated by the trapezoidal method within the last time point detected, and the remaining area was obtained as the ratio between the compound concentration detected at the last time point and the elimination constant (k_el_), which was obtained from the slope of the semilogarithmic plots (−slope × 2.3) [45]. The clearance (Cl) and distribution volume (V_d_) values were calculated according to the non-compartmental model as the ratios “dose/AUC” and “Cl/k_el_”, respectively, where k_el_ is the elimination constant obtained by the slope of semilogarithmic plot.

The absolute bioavailability values (F) referred to the orally administered free or formulated eugenol, cinnamaldehyde or D-limonene, were obtained as the ratio between the oral AUC and intravenous AUC values obtained for each compound in the bloodstream, normalized with respect to their doses, according to the following Equation (1) [46]:

(1) $$F=\frac{{\mathrm{AUC}}_{\mathrm{oral}}}{{\mathrm{AUC}}_{\mathrm{IV}}}\cdot \frac{{\mathrm{dose}}_{\mathrm{IV}}}{{\mathrm{dose}}_{\mathrm{oral}}},$$

The relative bioavailability values (RB) referred to the oral formulations of eugenol, cinnamaldehyde or D-limonene, with respect to the oral administration of free drugs were obtained as the ratio between the oral AUC values of formulated and free drug obtained in the blood stream for each compound, according to following Equation (2):

(2) $$\mathrm{RB}=\frac{{\mathrm{AUC}}_{\mathrm{formulated}}}{{\mathrm{AUC}}_{\mathrm{free}}}\cdot \frac{{\mathrm{dose}}_{\mathrm{free}}}{{\mathrm{dose}}_{\mathrm{formulated}}},$$

All the calculations were performed by using Graph Pad Prism software, version 7 (GraphPad Software Incorporated, La Jolla, CA, USA).

### 3.3. HPLC Analysis

The quantification of eugenol, cinnamaldehyde or D-limonene, was performed by HPLC. The chromatographic apparatus consisted of a modular system (model LC-10 AD VD pump and model SPD-10A VP variable wavelength UV−vis detector; Shimadzu, Kyoto, Japan) and an injection valve with 20 μL sample loop (model 7725; Rheodyne, IDEX, Torrance, CA, USA). Separations were performed at room temperature on a 5 μm Hypersil BDS C-18 column (150 mm × 4.6 mm i.d.; Alltech Italia Srl, Milan, Italy) equipped with a guard column packed with the same Hypersil material. The volume injection was 10 μL. Data acquisition and processing were performed on a personal computer using CLASS-VP Software, version 7.2.1 (Shimadzu Italia, Milan, Italy).

For the eugenol and cinnamaldehyde analysis the mobile phase consisted of an isocratic mixture of water and acetonitrile at a ratio of 50:50 (*v*/*v*); the flow rate was 1 mL/min. The detector was set at 210 nm or 290 nm for eugenol or cinnamaldehyde analysis, respectively. The retention times obtained were 3.2 min for cinnamaldehyde, 3.4 min for eugenol, and 5.4 min for carbazole used as internal standard for the quantification of eugenol or cinnamaldehyde in blood samples.

For the D-limonene analysis the mobile phase consisted of an isocratic mixture of water and acetonitrile at a ratio of 20:80 (*v*/*v*); the flow rate was 1 mL/min. The detector was set at 205 nm. The retention times obtained were 4.9 min for D-limonene and 8.4 min for GER-UDCA, obtained by the conjugation of geraniol with ursodeoxycholic acid [44] and used as internal standard for the D-limonene quantification in blood samples.

The chromatographic precision was evaluated by repeated analysis (*n* = 6) of the same sample solution containing 100 µM eugenol or D-limonene dissolved in a mixture of water and acetonitrile 25:75 (*v*/*v*), or 100 µM cinnamaldehyde dissolved in a mixture of water and acetonitrile 50:50 (*v*/*v*). For these three compounds the chromatographic precision, expressed as the relative standard deviation (RSD) value, was ≤0.89%. The calibration curves of peak areas versus concentration were generated in the range from 0.5 to 500 μM (eugenol: 0.082 μg/mL–82.11 μg/mL; cinnamaldehyde: 0.066 μg/mL–66.08 μg/mL; D-limonene: 0.068 μg/mL–68.12 μg/mL) for the compounds dissolved in their water and acetonitrile mixtures; over this range, the calibration curves were linear (*n* = 10, *r* ≥ 0.998, *p* < 0.0001).

A preliminary analysis performed on blank CSF and blood samples showed that their components did not interfere with retention times of eugenol, cinnamaldehyde, D-limonene and their internal standards (carbazole or GER-UDCA).

For CSF simulation, standard aliquots of balanced solution (Dulbecco’s phosphate buffer saline—DPBS—without calcium and magnesium) in the presence of 0.45 mg/mL BSA were used [47,48]. In this case, the chromatographic precisions were evaluated by repeated analysis (*n* = 6) of the same sample solution containing 2 μM of eugenol (0.33 μg/mL), cinnamaldehyde (0.26 μg/mL) or D-limonene (0.27 μg/mL) and are represented by RSD values ≤ 1.24. The calibration curves of peak areas versus concentration in CSF simulation fluid of the analytes were generated in the range of 0.3 to 30 μM for eugenol (0.05 to 4.92 μg/mL), cinnamaldehyde (0.04 to 3.96 μg/mL) and D-limonene (0.04 to 4.09 μg/mL), appearing linear (*n* = 8, *r* ≥ 0.997, *p* < 0.0001).

Recovery experiments from blood samples were performed comparing the peak areas extracted from blood test samples (10 μM, 1.64 µg/mL for eugenol, 1.32 µg/mL for cinnamaldehyde and 1.36 µg/mL for D-limonene) at 4 °C (*n* = 6) with those obtained by injection of an equivalent concentration of the analytes dissolved in water-acetonitrile mixture (50:50 *v*/*v* for cinnamaldehyde; 25:75 *v*/*v* for eugenol and D-limonene). The average recoveries ± SD were 98.2 ± 2.3% for eugenol, 44.6 ± 1.8% for cinnamaldehyde and 88.6 ± 2.9% for D-limonene. The concentrations of these compounds were therefore referred to as peak area ratio with respect to their internal standard (carbazole for eugenol and cinnamaldehyde or GER-UDCA for D-limonene). The precisions of the methods based on peak area ratio were represented by RSD values ≤ 1.24. The calibration curves referred to these compounds were constructed by using nine different concentrations in whole blood at 4 °C, ranging from 0.5 to 200 μM for eugenol (0.08 to 32.84 μg/mL), cinnamaldehyde (0.07 to 26.43 μg/mL) and D-limonene (0.07 to 27.25 μg/mL) and appeared linear (*n* = 9, *r* ≥ 0.995, *p* < 0.0001).

### 3.4. PC12 Cells Culture and Treatments

PC12 cell line (RRID:CVCL_0481), derived from rat adrenal gland pheochromocytoma, was a kind gift from Dr Federica Brugnoli from the Department of Translational Medicine, University of Ferrara, Ferrara (Italy). The cells were grown in RPMI-1640 (HiGlutaXL) medium (Microtech, Naples, Italy), supplemented with 100 µg/mL streptomycin and 100 IU/mL penicillin, 10% horse serum (HS), and 5% fetal bovine serum (FBS). PC12 cells were gently removed from the 4 µg/cm^2^ collagen IV-coated flask with a rubber scraper, transferred as cell suspension into a Falcon tube and resuspended through a sterile tip to avoid any clumping, then split into separate flasks twice weekly. To differentiate neuronal phenotypes, PC12 cells were seeded in 4 µg/cm^2^ collagen IV-coated 12-well plates at a density of 5000 cells/well, and they were then left to adhere to the well for 24 h. The next day, adhering cells were washed once with serum-free Dulbecco’s Modified Eagle’s Medium (DMEM) HiGlutaXL medium and then switched to DMEM HiGlutaXL medium, supplemented with 100 ng/mL nerve growth factor (NGF) and 1% HS up to 14 days of differentiation period, visually displayed by the high occurrence of axonal extensions. All the proliferative and differentiated cultures were maintained at 37 °C in a humidified 5% CO_2_ atmosphere.

### 3.5. Cell Viability Assay

PC12 cells, seeded at a density of 2500 cells/well, counted by using Scepter^TM^ 2.0 automated cell counter (Merck Millipore, Milan, Italy) and differentiated in collagen IV-treated 96-well plates, were assayed with the 3-(4,5-dimethylthiazol-2-yl)-2,5-diphenyltetrazolium (MTT) test for their viability during the incubation with increasing concentrations (2, 25, 50 and 100 µM) of eugenol in 0.2 mL DPBS, supplemented with 0.9 mM calcium, 0.5 mM magnesium and 5.3 mM glucose for 2 h at 37 °C in a 95% humidified atmosphere with 5% CO_2_. The 100% of cell viability was stated as control in the absence of compound. Then, incubation buffer was withdrawn, and 0.2 mL per well of a solution of MTT 500 µg/mL was added to each well and kept for 2 h at 37 °C in a humidified atmosphere at 95% with 5% CO_2_. The absorbance of each sample was measured with a multiplate reader spectrophotometer. After the conversion of the substrate to a chromogenic product by metabolically active cells, the MTT solution was removed, and the purple MTT formazan crystals were solubilized with 0.1 mL/well of DMSO for 60 min at 37 °C. The absorbance of each sample was measured with a multiplate reader spectrophotometer (Sunrise^®^ Microplate Reader, Tecan Trading AG, Männedorf, Switzerland) at 570 nm, using 690 nm as a reference wavelength. Cell viability, proportional to absorbance, was reported as ratio between the absorbance resulting from the treatments and absorbance resulting from the control, considered as 100% of viable cells. Data were plotted as graphs and analyzed using GraphPad software (Prism, version 7.0, San Diego, CA, USA).

### 3.6. Dopamine Release

PC12 cells differentiated to neuronal-like phenotype in collagen IV-treated 12-well plates, were first synchronized all to the same circadian phase and cell cycle by a serum shock (50% HS and 50% DMEM HiGlutaXL) for 2 h at 37 °C [49] to achieve a synchronous release of dopamine [50]. Synchronization medium was then aspirated off, cells were washed with 3 × 1 mL and equilibrated for 15 min at 37 °C in 1 mL of Krebs Ringer HEPES (KRH) physiological buffer, following the recipe of Mount et al. [40] (25 mM HEPES/Tris, pH 7.4, 1.2 mM KH_2_PO_4_, 125 mM NaCl, 4.8 mM KCl, 1.2 mM MgSO_4_, 2.2 mM CaCl_2_, 5.6 mM glucose, 1.0 mM ascorbic acid, 10 µM pargyline, 1.0 µM nomifensine). Basal or tonic pre-stimulation dopamine release was harvested, followed by the phasic release evoked by 60 mM K^+^ or eugenol in 1 mL KRH buffer, which is finally followed by the post-stimulation phase in 1 mL KRH for 5 min at 37 °C, which quantifies dopamine accumulation after the stimulation. All the three steps of dopamine release were collected sequentially during a time course of stimulation for 5, 15, 30, 60 and 120 min with 25 µM eugenol and for 5 min of stimulation with increasing concentrations of eugenol (1, 2, 5, 10 and 25 µM) together with 60 mM K^+^, as a positive control for functional dopamine release in PC12 cells. To remove cell debris, all samples of 1 mL incubation KRH at the end of treatments were centrifuged at 1000× *g* for 20 min at 4 °C and stored at −20 °C until dopamine levels were determined by ELISA assay.

### 3.7. Enzyme-Linked Immunosorbent Assay (ELISA)

Dopamine levels in 1 mL incubation KRH buffer were detected by means of an ELISA assay, based on the sandwich ELISA principle for detection of dopamine, following manufacturer’s instructions (IBL International, Hamburg, Germany–catalog no: RE59161; purchased from Tecan Italia S.r.I, Milan, Italy). After the substrate reaction, the intensity of the developed color was proportional to the amount of dopamine, detected at 450 nm using a microplate reader spectrophotometer (Sunrise^®^ Microplate Reader, Tecan Trading AG, Männedorf, Switzerland). Results of samples were determined directly using the standard curve. Results are presented as mean ± SE values of four independent experiments. Data were plotted and analyzed with GraphPad software (Prism, version 7.0, San Diego, CA, USA).

### 3.8. Statistical Analysis

Statistical comparisons between AUC values or absolute bioavailability values or relative bioavailability values were performed by one-way analysis of variance (ANOVA) followed by Dunnett’s post-test, or by t test. Statistical analysis referred to data from cell viability and dopamine release was performed by one-way ANOVA, followed by Bonferroni’s multiple comparisons test. Significance was set at *p* < 0.05. All the calculations were performed by using Graph Pad Prism software, version 7 (GraphPad Software Incorporated, La Jolla, CA, USA).

## 4. Conclusions

The main findings of this study are the following: (*i*) easily achievable HPLC-UV methods have been developed to quantify eugenol, cinnamaldehyde and D-limonene, in the bloodstream, after appropriate purification procedures of blood samples; moreover, CSF withdrawn by means of cisternal puncture allowed us to couple CSF and blood profiles of each compound; (*ii*) relatively poor oral bioavailability characterized these compounds, ranging from 4% for eugenol to 7% for cinnamaldehyde and D-limonene; (*iii*) eugenol demonstrated the highest aptitude to permeate the CNS after both intravenous and oral administration; (*iv*) despite the significant decrease of oral bioavailability obtained by formulations designed to reduce the eugenol intestinal absorption, eugenol in the CSF of rats was still significantly detectable; (*v*) eugenol was significantly efficacious to stimulate in vitro neuronal cell viability, and induced a time- and dose-dependent release of dopamine, also at the concentrations reached in the CSF after oral administration.

In terms of the future, eugenol appears as a potential therapeutic agent for the prevention and/or treatment of neurogenerative disorders, following its oral administration.

This study has, however, some potential limitations that should be taken in consideration. First of all, there are no data on the possible mechanism(s) of action by which eugenol modulates PC12 cell viability and dopamine release. Furthermore, it remains to be elucidated whether eugenol is able to increase brain dopamine levels in vivo. Finally, although we suggest a potential use of EO component (eugenol) formulations for chronic daily oral administrations deputed to the microbiota maintenance, the beneficial effects of these formulations on gastrointestinal microbiota composition, remain to be proved. All these aspects need to be investigated in further studies to support the potential therapeutic use of eugenol against neurodegenerative diseases.

## Figures and Tables

**Table 1:** Pharmacokinetic parameters referred to the bloodstream of eugenol, cinnamaldehyde and D-limonene after intravenous administration to rats. Data are reported as mean ± SE (*n* = 4). C_0_: concentration at the end of infusion; k_el_: pseudo first order kinetic elimination rate constant; t_1/2_: half-life; AUC: area under concentration from the starting time of infusion to infinity; Cl: clearance; V_d_: volume of distribution.

Compound	Dose	C_0_ (μg/mL)	k_el_ (min^−1^)	t_1/2_ (min)	AUC (μg mL^−1^·min)	Cl (mL·min·kg^−1^)	V_d_ (mL·kg^−1^)
Eugenol	5 mg (20 mg/kg)	16.5 ± 0.2	0.036 ± 0.002 ^a^	19.4 ± 2.1 ^a^	174.8 ± 3.1	114 ± 2 ^b^	3212 ± 247 ^b^
Cinnamaldehyde	5 mg (20 mg/kg)	20.3 ± 1.5	0.030 ± 0.001	23.1 ± 1.6	506.0 ± 22.3	39.5 ± 1.7	1320 ± 190
D-Limonene	25 mg (100 mg/kg)	19.5 ± 0.4	0.056 ± 0.004	12.4 ± 0.9	352.2 ± 13.1	284 ± 10	5516 ± 558

^a^ terminal; ^b^ mean.

**Table 2:** Pharmacokinetic parameters referred to cerebrospinal fluid (CSF) of eugenol, cinnamaldehyde and D-limonene after intravenous administration to rats. Data are reported as mean ± SE (*n* = 4). C_max_: maximum concentration obtained in CSF; T_max_: time of C_max_; AUC: area under concentration from the end of infusion to last time of detection; R: ratio of concentration between CSF and bloodstream at T_max_.

Compound	Dose	C_max_ (μg/mL)	T_max_ (min)	AUC (μg·mL^−1^·min)	R
Eugenol	5 mg (20 mg/kg)	2.79 ± 0.18	10	56.1 ± 4.2	0.96 ± 0.08
Cinnamaldehyde	5 mg (20 mg/kg)	2.04 ± 0.36	10	96.7 ± 11.0	0.16 ± 0.04
D-Limonene	25 mg (100 mg/kg)	0	0	0	0

**Table 3:** Pharmacokinetic parameters referred to the bloodstream of eugenol, cinnamaldehyde and D-limonene after oral administration to rats. Data are reported as mean ± SE (*n* = 4). C_max_: maximum concentration obtained in the bloodstream; T_max_: time of C_max_; AUC: area under concentration from time 0 to infinity; F: absolute bioavailability; RB: relative bioavailability.

Formulation	Dose	C_max_ (μg/mL)	T_max_ (min)	AUC (μg·mL^−1^·min)	F (%)	RB (%)
Free eugenol	125 mg (500 mg/kg)	3.4 ± 0.2	10	185.7 ± 3.1	4.25 ± 0.11	100
EU-GN	125 mg (500 mg/kg)	1.29 ± 0.10	10	95.6 ± 1.5 ^a^	2.19 ± 0.05	51.5 ± 1.1 ^c^
EU-CD	125 mg (500 mg/kg)	0.35 ± 0.05	10	31.6 ± 1.7 ^a^	0.72 ± 0.04	17.0 ± 1.0 ^c^
EU-SL	125 mg (500 mg/kg)	0.23 ± 0.01	20	29.2 ± 0.7 ^a^	0.67 ± 0. 02	15.7 ± 0.5 ^c^
Free cinnamaldehyde	100 mg (400 mg/kg)	8.36 ± 0.49	40	742.7 ± 18.4	7.33 ± 0.37	100
AC-GN	100 mg (400 mg/kg)	3.71 ± 0.64	40	278.3 ± 15.9 ^b^	2.75 ± 0.19	37.5 ± 3.2 ^d^
Free D-limonene	50 mg (200 mg/kg)	2.31 ± 0.44	30	49.6 ± 6.7	7.04 ± 0.96	100
LM-CA	50 mg (200 mg/kg)	0	0	0	0	0

^a^*p* < 0.001 versus free eugenol; ^b^ *p* < 0.001 versus free cinnamaldehyde; ^c^ with respect to free eugenol; ^d^ with respect to free cinnamaldehyde.

**Table 4:** Pharmacokinetic parameters referred to cerebrospinal fluid (CSF) of eugenol, cinnamaldehyde and D-limonene after oral administration to rats. Data are reported as mean ± SE (*n* = 4). C_max_: maximum concentration obtained in CSF; T_max_: time of C_max_; AUC: area under concentration from the end of infusion to last time of detection.

Compound	Dose	C_max_ (μg/mL)	T_max_ (min)	AUC (μg·mL^−1^·min)
Free eugenol	125 mg (500 mg/kg)	0.89 ± 0.06	20	30.97 ± 2.18
EU-GN	125 mg (500 mg/kg)	0.39 ± 0.03	20	11.05 ± 0.69 ^a^
EU-CD	125 mg (500 mg/kg)	0.146 ± 0.002	20	5.96 ± 0.31 ^a^
EU-SL	125 mg (500 mg/kg)	0.110 ± 0.006	20	5.69 ± 0.09 ^a^
Free cinnamaldehyde	100 mg (400 mg/kg)	0	0	0
AC-GN	100 mg (400 mg/kg)	0	0	0
Free D-limonene	50 mg (200 mg/kg)	0	0	0
LM-CA	50 mg (200 mg/kg)	0	0	0

^a^*p* < 0.001 versus free eugenol.

**Table 5:** Doses and administration modalities to rats of eugenol, cinnamaldehyde and D-limonene by intravenous and oral ways.

**Intravenous administration**				
**Compound**	**Dose**	**Formulation**	**Administered volume**	**Infusion time**
Eugenol	5 mg (20 mg/kg)	12.5 mg/mL eugenol emulsion	0.4 mL	2 min
Cinnamaldehyde	5 mg (20 mg/kg)	12.5 mg/mL cinnamaldehyde emulsion	0.4 mL	2 min
D-Limonene	25 mg (100 mg/kg)	Pure compound	30 µL	2 min
**Oral administration**				
**Compound**	**Dose**	**Formulation**	**Administration method**	**Administered amounts**
Eugenol (free)	125 mg (500 mg/kg)	Corn oil solution (125 mg/mL)	Gavage	1 mL
Eugenol	125 mg (500 mg/kg)	EU-GN (20% *w*/*w*)	Palatable food	625 mg
Eugenol	125 mg (500 mg/kg)	EU-CD (30 mg/mL)	Gavage	4.2 mL
Eugenol	125 mg (500 mg/kg)	EU-SL (390 mg/mL)	Gavage	390 μL
Cinnamaldehyde (free)	100 mg (400 mg/kg)	Corn oil solution (100 mg/mL)	Gavage	1 mL
Cinnamaldehyde	100 mg (400 mg/kg)	AC-GN (14% *w*/*w*)	Palatable food	714 mg
D-Limonene (free)	50 mg (200 mg/kg)	Corn oil solution (100 mg/mL)	Gavage	500 μL
D-Limonene	50 mg (200 mg/kg)	LM-CA (14% *w*/*w*)	Palatable formulation	300 mg

## Data Availability

Not applicable.
